# Evaluation of New Biphasic Calcium Phosphate Bone Substitute: Rabbit Femur Defect Model and Preliminary Clinical Results

**DOI:** 10.1007/s40846-016-0203-3

**Published:** 2017-01-19

**Authors:** Yeong-Jang Chen, Jwo-luen Pao, Chiang Sang Chen, Yu-Chun Chen, Chun-Chien Chang, Fang-Ming Hung, Chih-Hung Chang

**Affiliations:** 1 Department of Orthopaedic Surgery, Far Eastern Memorial Hospital, Banqiao, 21 Nanya S. Rd. Sec. 2, Banciao Dist., New Taipei City, 220 Taiwan; 2 Department of Orthopaedic Surgery, National Taiwan University Hospital, Taipei, 10002 Taiwan; 3 Institute of Biomedical Engineering, National Taiwan University, Taipei, 10002 Taiwan; 4 Department of Surgical Intensive Care Unit, Far Eastern Memorial Hospital, New Taipei City, 220 Taiwan; 5 Graduate School of Biotechnology and Bioengineering, Yuan Ze University, Taoyuan, 32003 Taiwan

**Keywords:** Bone substitute, Biphasic calcium phosphate, Fracture healing, Hydroxyapatite

## Abstract

Autogenous bone grafting, used to repair bone defects, is limited and the donor site can experience complications. Compared to autogenous bone graft, artificial bones have different porosity, which might make them suitable alternatives to bone grafts. Here, two porous biphasic calcium phosphate bone substitutes, namely Bicera™ and Triosite™, are used in an animal study and clinical practice to find a suitable porosity for implantation. Bicera™ and Triosite™ consist of 60 wt% hydroxyapatite and 40 wt% β-tricalcium phosphate, with the porosity of Bicera™ (82%) being higher than that of Triosite™ (70%). In the animal study, the implantation procedure was carried out on twenty-four female New Zealand rabbits. 12 weeks after implantation, the new bones were well infiltrated into the Bicera™ and Triosite™ bone grafts. In the clinical study, patients with comminuted fracture, fracture nonunion, or arthrodesis were included in the study of bone substitution with Bicera™. 27 patients underwent fracture fixation treatment. Bone healing of 22.22% (6/27) of patients happened within 3 months after the surgery, and that of 66.67% (18/27) of patients happened within 6 months. These results reveal that Bicera™ has good incorporation with host bone, and that new bone is able to grow within the porous structure, giving it high potential in the treatment of bone defects.

## Introduction

Treatment for comminuted fracture is a challenging task. This type of fracture usually occurs with osteoporosis or major trauma. Comminuted fractures usually have bone defects, which breakdown the bone structure and reduce potential of bone healing. In order to promote bone healing, bone substitute is often used to provide a scaffold for the new bone to regenerate or to support the articular fragments. Although autogenous bone grafting is favorable for bone defect treatment, it still has some serious complications such as limited graft volume and donor site morbidities [1, 2]. Therefore, the allograft and xenograft are frequently used as an alternative treatment. In recent decades, a variety of artificial bone substitutes have been developed for clinical use [3].

Synthetic calcium phosphates are commonly used in bone substitutes [4]. Within the group of phosphates, hydroxyapatite (HA) and tricalcium phosphate (TCP) are the most common materials used for bone substitution [5]. HA has excellent biocompatibility with the bony environment; however, it is mostly used for surface coating on metal implants due to its low solubility and brittle nature [6]. TCP has the advantage of osteoconductivity, but it is limited in clinical use due to its rapid degradation rate [7]. Therefore, biphasic calcium phosphate (BCP) has been used to overcome the disadvantages of HA and TCP. Since BCP includes HA and TCP, it provides optimal dissolution and good bioactivity, cell attachment, proliferation, and differentiation for new bone regeneration [8].

Although previous studies have shown that bone substitute porosity plays a significant role in bone regeneration, the optimal porosity for bone healing is not known [9]. The present study thus investigates the most suitable biodegradable porous artificial bone using the rabbit model and clinical trials. Two kinds of porous BCP bone substitute are evaluated, namely Bicera™ and Triosite™. Bicera™ is composed of 60% HA and 40% TCP and has a pore size of about 461.92 ± 90.66 μm. It is characterized by interconnected pores with a porosity of 82.62 ± 0.02%. Triosite™ has the same composition, but its pore size and porosity are approximately 450 μm and 70%, respectively (Table 1). The objective of the present study is to investigate the Bicera™ bone substitute for comminuted fracture because of its potential to provide a highly porous and interconnected structure for new bone regeneration. Specifically, post-operative healing after treatment with BCP bone substitute is demonstrated. The clinical outcomes of 27 patients in a retrospective study on BCP bone substitute are also described. Additionally, histological confirmation of the incorporation of Bicera™ and Triosite™ and the formation of new bone are evaluated using the rabbit model with a femoral condyle cancellous bone defect.

**Table 1:** Comparison of Bicera™ and Triosite™

	Bicera™	Triosite™
Composition	Biphasic calcium phosphate	Biphasic calcium phosphate
Porosity (%)	82.62 ± 0.02	70
Pore size (μm)	461.92 ± 90.66	450 ± 49

## Materials and Methods

### Materials

The bone substitutes used in the study are Bicera™ (Wiltrom Ltd, Taiwan.) and Triosite™ (Zimmer Ltd., Swindon, UK). Bicera™ and Triosite™ are compossed of BCP (60% HA and 40% TCP). Information on Bicera™ and Triosite™ is presented in Table 1. Bicera™ has a higher porosity and similar pore size compared to those of Triosite™. The interconnected pores of Bicera™ can act like a guide for bone tissue growth. Both BCP bone substitutes used in this study are FDA-approved in the USA as bone void filler.

### Animal Study

The animal study was carried out using twenty-four female New Zealand rabbits (20 weeks old, average weight of approximately 3.0 kg) based on a protocol approved by the Institutional Animal Experiment Committee of National Taiwan University, Taipei, Taiwan.

**Fig. 1:**
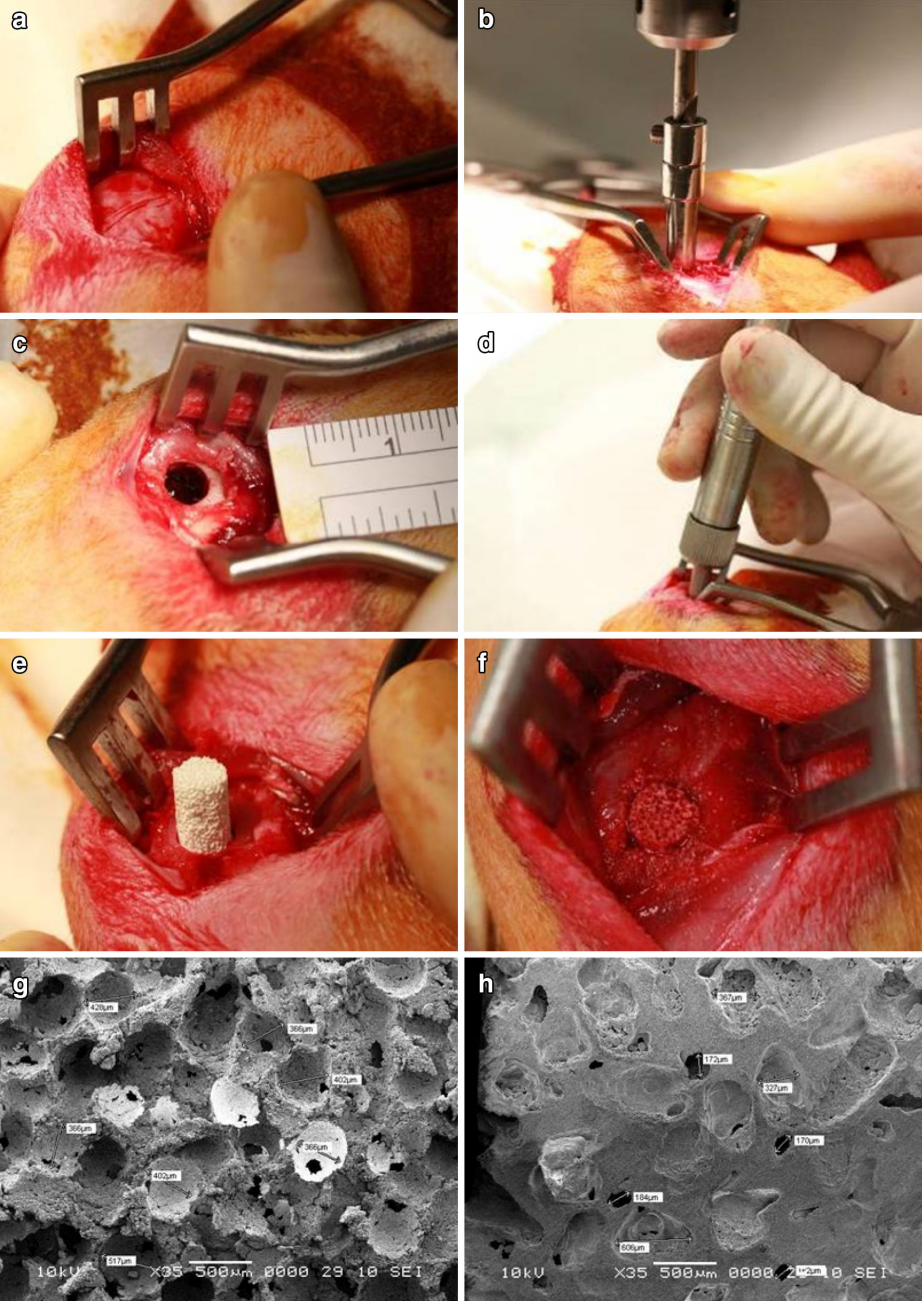
Implantation procedure **a** Exposure of lateral femoral condyle. **b** Bony defect 5 mm in diameter and 10 mm in length was created in both femurs using a surgical drill bit. **c**, **d** Diameter and depth of the defect were measured. **e**, **f** Bone substitute 5 mm in diameter and 10 mm in length was filled into the defect site. SEM micrographs of **g, h** Bicera™ and Triosite™

The rabbits were anesthetized by an intramuscular injection of a mixture of Ketamin (0.5 ml/kg) and Comblene (0.5 ml/kg). The bone graft substitutes (Bicera™ and Triosite™), 5 mm in diameter and 10 mm in length, were sterilized prior to implantation. A 2.5-cm skin incision was made to expose the lateral femoral condyle of the thigh. A bony defect 5 mm in diameter and 10 mm in depth was created on both femurs by drilling using a stopper set designed for creating critical size defects. The diameter and depth of the defect were measured using a sterilized ruler and a depth gauge, respectively, to confirm the desired dimension. Bone substitute (5 mm in diameter and 10 mm in length) was used to fill in the defect site (Fig. 1). Finally, the endodermis, muscle tissue, and surface skin were closed with sutures after implantation.

Bicera™ was implanted into all twenty-four left femurs. Triosite™ was placed into twelve right femurs, with the remaining twelve right femurs left cavernous as the empty control. The femoral defects were evaluated at 4, 8, and 12 weeks following surgery.

Animals were sacrificed at 4, 8, and 12 weeks. Femur samples were harvested at the designated time period and fixed in 10% formalin for 24 h. For histological analysis, specimens were embedded in polymethyl methacrylate. These specimens were cut and aligned with the diametral plane. The section was mounted on a slide and stained with Masson-Goldner trichrome. After staining, muscle fiber was red, collagen was blue, and voids were white. The toluidine blue method was used to identify mineralized bone. After staining with toluidine blue, mineralized bone was blue. The sections were also collected for histomorphometric analysis.

### Human Clinical Study

From June, 2011 to December, 2012, 27 patients with comminuted fracture (scaphoid, tibia, humerus, clavicle, femur, wrist, distal radius, or ankle) were treated with Bicera™. The artificial bone grafts were placed between the broken bone (e.g., for a humeral shaft fracture) or at the subcortical area to fill the space (e.g., for a tibial plateau fracture). The amount of bone substitute depended on the bone defect; up to 5 ml of Bicera™ was used intraoperatively. All procedures were performed at Far Eastern Memorial Hospital, New Taipei City, Taiwan. This study evaluated the radiographs monthly. Fracture healing or bone fusion was defined as callus bridging with at least three cortices in the radiographs. The duration from operation to fracture healing or bone fusion was recorded.

Inclusion criteria for patients were comminuted fractures, nonunion, or arthrodesis of four limbs. Exclusion criteria were an age below 80 years and having concomitant diseases that might be worsened by invasive treatment of the fracture, such as a local tumor. Patients were also excluded if they had a history of malignancy, infection, abnormal laboratory finding, a liver function abnormality, or metabolic bone disease. Patients that met the inclusion criteria were offered the procedure with bone substitutes and informed consent was obtained. The clinical study was approved by the Research Ethics Review Committee (103053-E) of Far Eastern Memorial Hospital, New Taipei City, Taiwan.

## Results

### Radiographic Evaluation in Animal Study

**Fig. 2:**
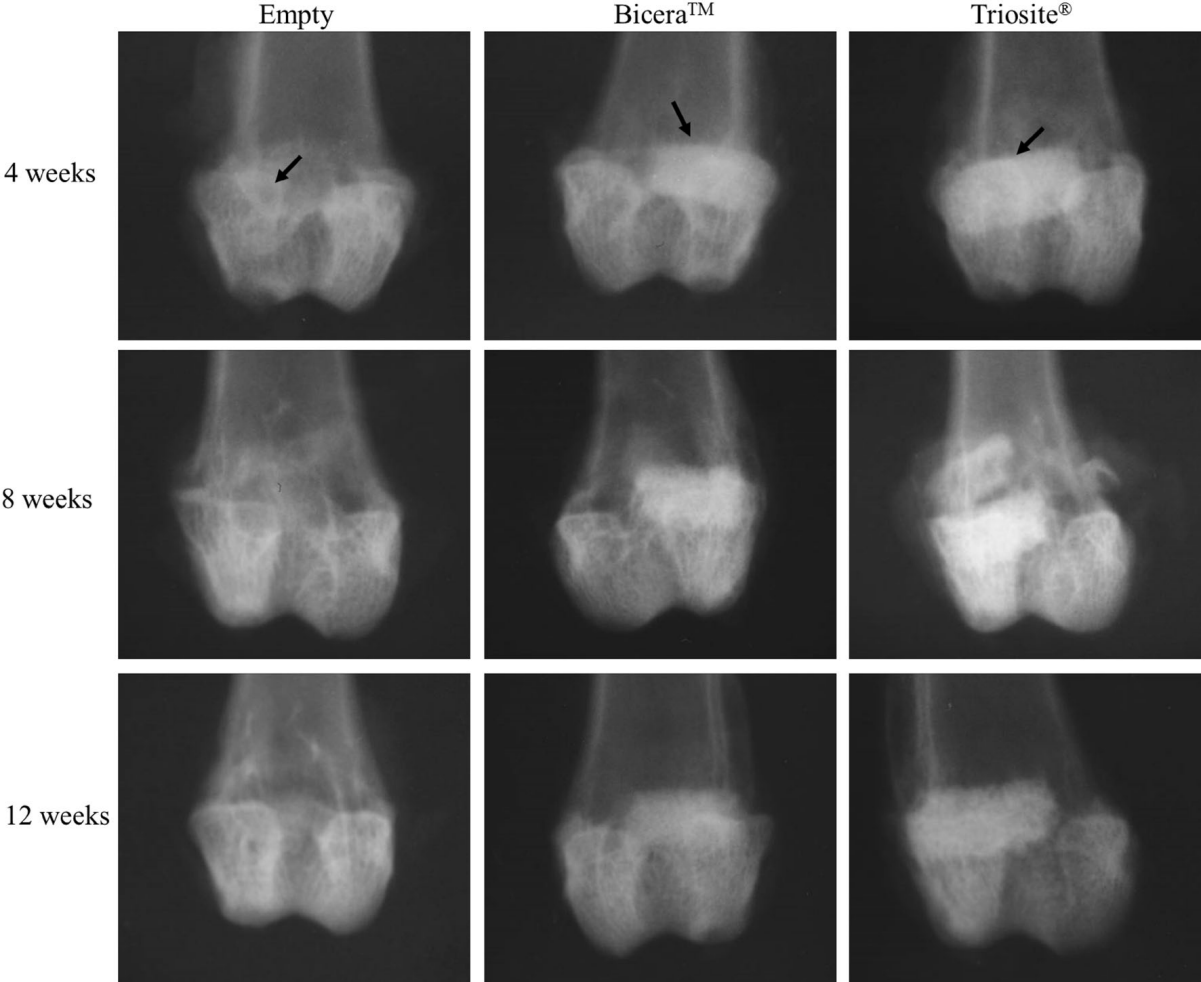
Rabbit femur condyle X-ray image. Defect without bone substitute was still an empty cavity. Both bone substitutes remained completely intact, with no significant degradation observed 12 weeks after implantation (*arrow* implant site)

From Table 1 and scanning electron microscopy (SEM) micrographs (Fig. 1), Bicera™ had superior interconnected pore structure and higher porosity compared to those of Triosite™. The radiographs of implant sites at 4, 8, and 12 weeks after implantation showed that both bone substitutes were well incorporated with surrounding bone (Fig. 2). The defect of the lateral femoral condyle without bone substitute was still empty. 12 weeks after implantation, both bone substitutes showed no significant degradation.

### Histological Examination in Animal Study

**Fig. 3:**
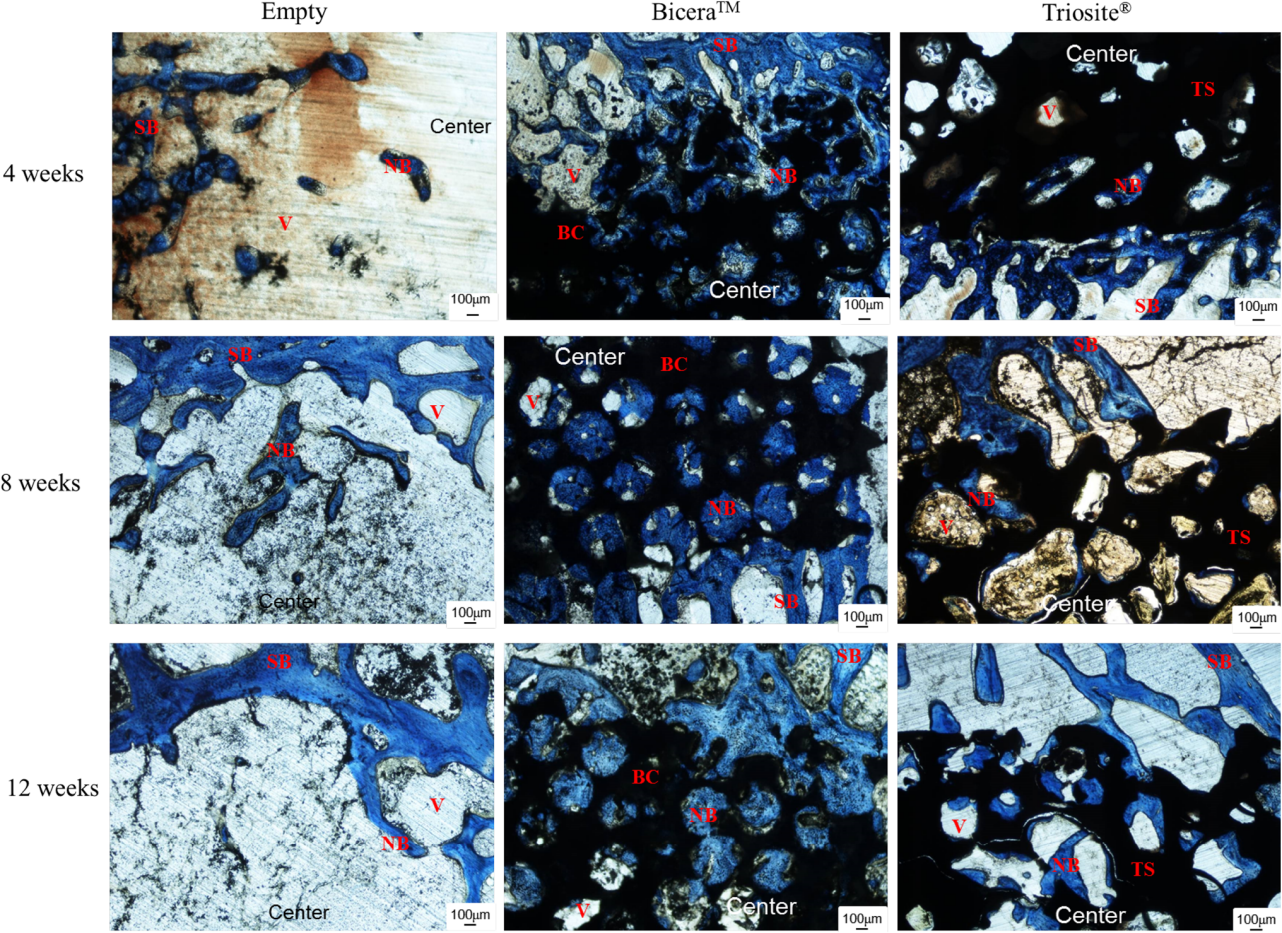
Histological image of rabbit femoral condyle tissue section. In Bicera™ group, new bone (*blue area*) grew into porous structure of bone substitute (*black area*). In Triosite™ group, there was an obvious interface between bone substitute (*black area*) and host bone (*blue area*) (*SB* surrounding bone, *NB* new bone, *BC* Bicera™, *TS* Triosite™, *Center* central site in defect)

**Fig. 4:**
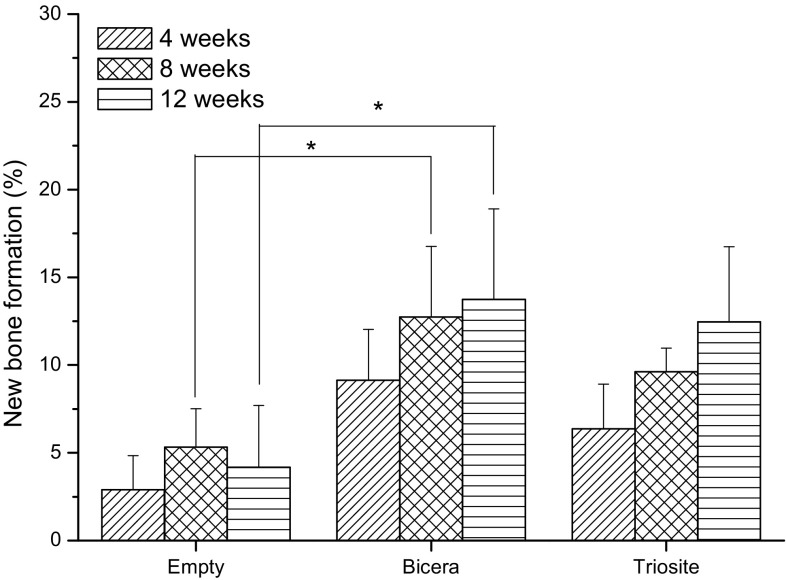
Percentage of new bone formation. 12 weeks after implantation, new bone formation occurred in up to 14% of total cavities in Bicera™. With Triosite™, new bone formation occurred in 12% of total cavities inside the material (*p ≤ 0.05)

The optical micrographs of the femoral condyle specimen are shown in Fig. 3. Four weeks after implantation, the defects were surrounded by bone tissue. The amounts of new bone that had formed in two bone substitute groups were significantly higher than that for the empty control. In the Bicera™ group, new bone had grown from the margin of macropores toward the inside of the substitute, whereas in the Triosite™ group, new bone formation occurred only around the periphery. 8 weeks after implantation, although the new bone grew in bone substitute groups, unlike in the Bicera™ group, void spaces were not filled completely in the Triosite™ group (Fig. 3). In the empty control group, defects were surrounded by bone tissue but failed to form any new bone. The porous structure of Bicera™ showed some sign of degradation. New bone was also observed inside the porous structure of Bicera™. In the Triosite™ group, the ingrowth of new bone occurred from the periphery toward the center of the defect site. At 12 weeks post-implantation, no fibrous tissue or inflammation response was observed at any defect site. The empty control group revealed no sign of bone growth at any defect site (Fig. 3). In Fig. 4, the area of new bone formation occupied only 4% of the cavities in the empty control group. The new bone formation occupied 14 and 12% of the area in the Bicera™ and Triosite™ groups, respectively. The two bone substitutes had similar bone formation properties. From the statistical analysis, there was a significant difference in new bone formation between the Bicera™ and empty control groups at 8 and 12 weeks. This indicates that Bicera™ had good osteoconductivity and that its high porosity served as a good scaffold structure for new bone ingrowth.

### Human Clinical Study

**Fig. 5:**
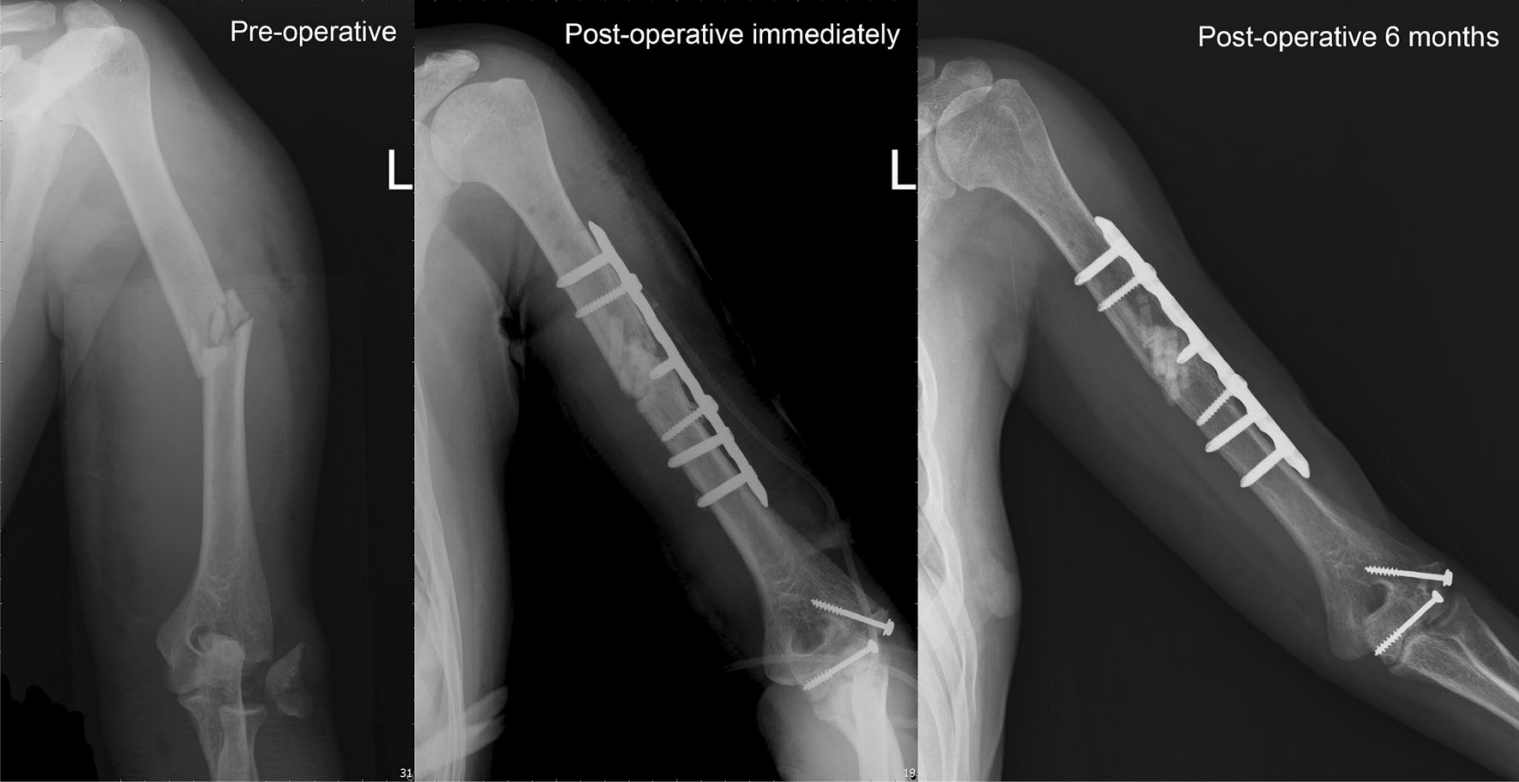
A 40-year-old women had left humeral shaft fracture. She underwent open reduction internal fixation and Bicera™ was filled into fracture (comminuted fracture). Radiograph revealed fracture union 6 months after operation

**Fig. 6:**
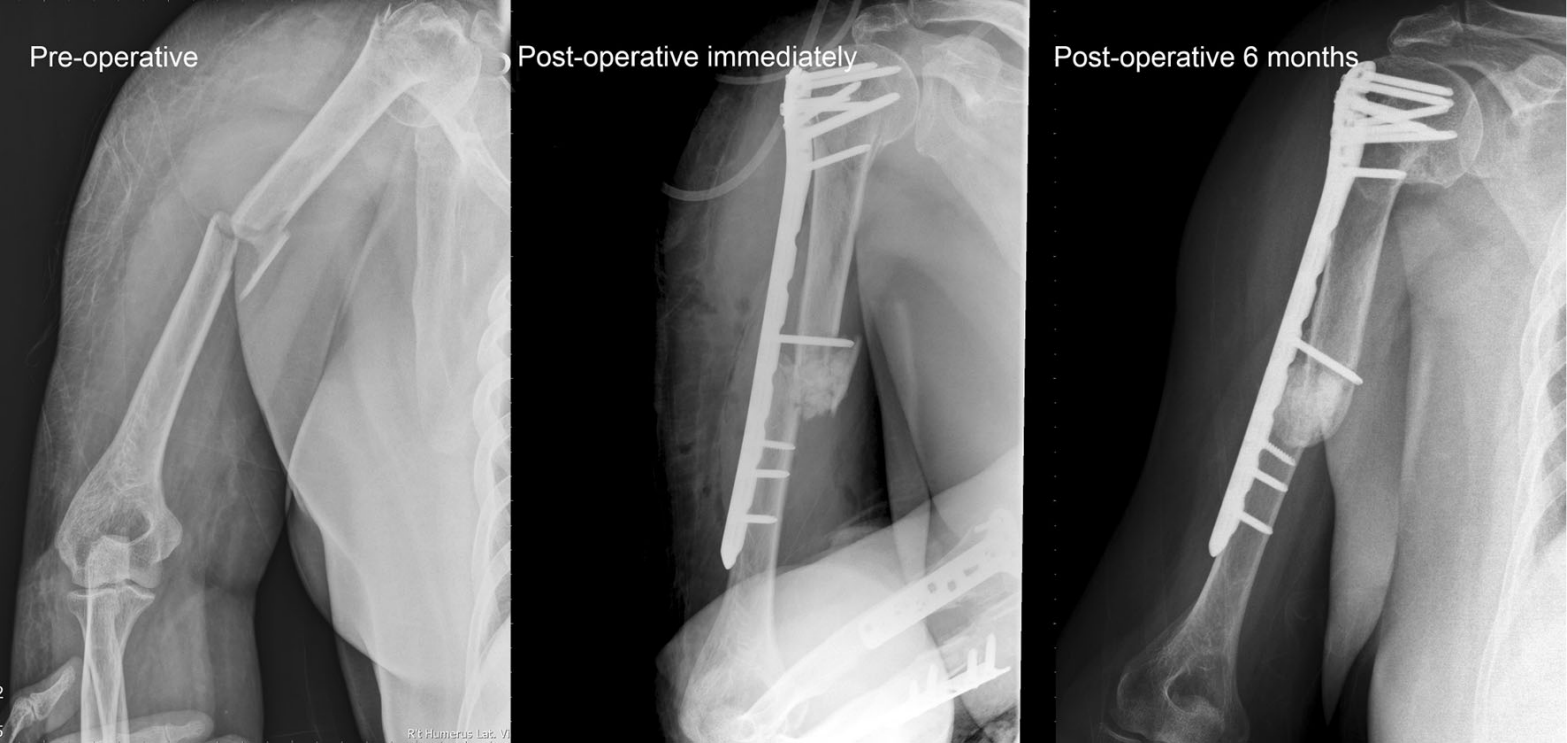
A 66-year-old women had right humeral shaft and neck fracture. She underwent open reduction internal fixation via a minimally invasive technique and Bicera™ was filled into fracture. Radiograph revealed fracture union 6 months after operation

A total of 44 patients were recruited according to the inclusion criteria. Fifteen patients were excluded: one had an infection, one had mal-union, three had poor radiograph quality, and ten had follow-up of less than 6 months. Of the remaining 27 patients (Table 2), 14 were men and 13 were women. The mean age was 44.22 years (range of 18–79 years). The numbers of revision fracture fixations, comminuted fracture fixations, and arthrodeses were 3, 23, and 1, respectively. Among the 27 patients, 6 patients had healed within 3 months, 12 patients had healed in 4–6 months, 2 patients had healed in 7–12 months, and 2 patients had delayed healing (16 and 18 months). The radiographs of patients who had bone union showed solid union (Figs. 5 and 6), and no further revision surgeries were needed. Of the five patients who failed to get bone union, two patients had hypertrophic nonunion, two had loss of reduction and further collapse, and one had radiolucent gaps in the final radiographs.

**Table 2:** Patient information

No.	Age (years)	Gender	Diagnosis	Union	Time to union (months)	Duration of follow-up (months)
1	26	F	Scaphoid fracture	O	16	16
2	56	M	Tibial medial plateau fracture	O	5	10
3	49	F	Proximal humeral fracture	O	3	4
4	62	F	Clavicle fracture s/p ORIF, loss of reduction	O	4	7
5	57	M	Right proximal tibia fracture	X		16
6	24	M	Distal femur fracture	O	18	18
7	30	M	Humeral shaft fracture s/p ORIF, implant failure	X		9
8	32	M	Tibial plateau fracture	O	2	18
9	46	F	Bilateral wrist rheumatoid arthritis	O	3	7
10	40	F	Humeral shaft open fracture	O	6	8
11	31	M	Clavicle shaft fracture	O	9	11
12	37	M	Tibia plateau fracture	O	8	18
13	79	F	Distal radius fracture	O	4	5
14	36	M	Left tibia plateau fracture	O	3	14
15	25	M	Femur proximal shaft fracture	X		15
16	32	M	Clavicle fracture	O	6	12
17	54	F	Tibia plateau fracture	O	4	14
18	33	F	Distal radius fracture	O	3	11
19	39	M	Distal radius fracture	O	4	12
20	70	F	Distal femur periprosthetic fracture	O	6	26
21	18	M	Clavicle fracture	O	5	8
22	27	M	Femoral shaft fracture	X		13
23	29	F	Tibia plateau fracture	O	5	15
24	48	M	Ankle bimalleolar fracture	O	3	4
25	66	F	Distal radio-ulnar fracture	O	6	8
26	74	F	Proximal tibia fracture	X		13
27	74	F	Supracondylar femoral fracture	O	6	13

*ORIF* open reduction and internal fixation, *s/p* status post

## Discussion

The architecture of ceramic bone substitutes is the key to creating an environment for osteogenic cell adhesion, proliferation, and differentiation. BCP (Bicera™ and Triosite™) is composed of two components, namely HA and TCP, which are the two most important calcium phosphate ceramics for bone regeneration [5]. HA resembles the inorganic component of human bone and is considered as an osteoconductive and bioactive ceramic [10]. TCP is gradually replaced by new bone due to its bioresorbability [11]. BCP ceramics have a longer degradation rate than that of TCP and are stable enough to provide bone formation for six months after implantation [12]. We conducted the rabbit femoral defect experiment to evaluate the effectiveness of the bone substitutes. The control groups were an empty defect and a defect filled with the commercial ceramic Triosite™.

In order to simulate the repair of bone with bone substitutes, we defined a critical defect 5 mm in diameter and 10 mm in length, which was drilled at the bilateral femoral condyles and filled with the same-size substitute. In this study, there was only a slight amount of new bone formation 12 weeks after implantation for the no treatment control group. Both bone substitutes exhibited different results based on radiographic and histological examination. At 4 weeks post-implantation, new bone was found in the macropores in Bicera™, and bone mineralization was found around the central area (Fig. 3). With Triosite™, new bone was discovered along the periphery, and there were only small amounts of new bone in the central region. 8 weeks after implantation, new bone infiltrated from the periphery toward the center for both bone substitutes. The porous structure of the substitutes plays an important role in osteoconductivity. At 12 weeks post-implantation, there was no evidential degradation of the ceramics. Both substitutes maintained almost complete structure for new bone ingrowth.

The primary goal of trauma treatment is the successful union of the bone. In this study, for the 27 patients with the bone substitute, bone fusion or union was evaluated based on radiographs. Callus bridging with at least three cortices in the post-operative radiographs was considered as bone fusion or union. In addition to bone fusion or union, the general purpose of fracture treatment is to stabilize the fracture and repair bone in its original position. Nonunion and delayed union are common complications of comminuted fracture and arthrodesis [13]. Bone substitute is a great way to speed up the healing process. In our preliminary clinical data, 22.22% (6/27) of patients who used Bicera™ had their bone fused within 3 months after the surgery, and 66.67% (18/27) of patients had their bone fused within 6 months. In a previous study, the healing time of bone defects for Triosite™ was more than 6 months [14]. Bicera™ led to a relatively faster healing time compared to that obtained with Triosite™ in the literature.

Pore size, structure, and porosity are factors influencing osteoinductivity, which induces cell differentiation to achieve osteogenesis [15, 16]. The higher porosity and more suitable pore size an interconnected pore structure has, the more bone cell transforms into new bone tissue [17]. In addition, a steady and gradual dissolution of BCP ceramics can help create a localized environment rich in calcium and phosphorus for osteogenic precursor cell adhesion, differentiation, production of bone matrix, and finally, ossification. The fast and sustained bone fusion observed in this study demonstrates that the porous BCP bone substitute has sufficient osteoconduction to help the fracture healing process.

Extensive animal and clinical studies have shown that porous implants can result in ingrown bone and bone regeneration [18–20]. Our animal study and clinical observation showed new bone infiltrating into Bicera™ 12 weeks after the implantation. Bicera™ exhibited relatively greater bone regeneration on the lateral femoral condyle compared to that of the control group. Bicera™ has interconnected pores with high porosity and induces osteoconduction for new bone formation.

## Conclusion

This study compared BCP bone substitutes. At 12 weeks post-implantation in rabbits, Bicera™ and Triosite™ bone substitutes had good incorporation with host bone, and new bone formation infiltrated into the porous structure. In clinical application, Bicera™ bone substitute has good potential for the treatment of bone defects (e.g., comminuted fracture or arthrodesis).
